# Hierarchical imaging: a new concept for targeted imaging of large volumes from cells to tissues

**DOI:** 10.1186/s12860-016-0122-8

**Published:** 2016-12-12

**Authors:** Irene Wacker, Waldemar Spomer, Andreas Hofmann, Marlene Thaler, Stefan Hillmer, Ulrich Gengenbach, Rasmus R. Schröder

**Affiliations:** 1 Cryo Electron Microscopy, Centre for Advanced Materials, Universität Heidelberg, Im Neuenheimer Feld 267, D-691120 Heidelberg, Germany; 2 HEiKA, Heidelberg Karlsruhe Research Partnership, Heidelberg, Germany; 3 Institute for Applied Computer Science, Karlsruhe Institute of Technology (KIT), Karlsruhe, Germany; 4 Carl Zeiss Microscopy GmbH, Carl-Zeiss Str. 22, D-73447 Oberkochen, Germany; 5 Electron Microscopy Core Facility, Universität Heidelberg, Im Neuenheimer Feld 345, D-69120 Heidelberg, Germany; 6 Cryo Electron Microscopy, CellNetworks, BioQuant, Universitätsklinikum Heidelberg, Im Neuenheimer Feld 267, D-691120 Heidelberg, Germany

**Keywords:** Array tomography, Serial sectioning, Section libraries, Hierarchical imaging, Large volume 3D reconstruction

## Abstract

**Background:**

Imaging large volumes such as entire cells or small model organisms at nanoscale resolution seemed an unrealistic, rather tedious task so far. Now, technical advances have lead to several electron microscopy (EM) large volume imaging techniques. One is array tomography, where ribbons of ultrathin serial sections are deposited on solid substrates like silicon wafers or glass coverslips.

**Results:**

To ensure reliable retrieval of multiple ribbons from the boat of a diamond knife we introduce a substrate holder with 7 axes of translation or rotation specifically designed for that purpose. With this device we are able to deposit hundreds of sections in an ordered way in an area of 22 × 22 mm, the size of a coverslip. Imaging such arrays in a standard wide field fluorescence microscope produces reconstructions with 200 nm lateral resolution and 100 nm (the section thickness) resolution in z.

By hierarchical imaging cascades in the scanning electron microscope (SEM), using a new software platform, we can address volumes from single cells to complete organs. In our first example, a cell population isolated from zebrafish spleen, we characterize different cell types according to their organelle inventory by segmenting 3D reconstructions of complete cells imaged with nanoscale resolution. In addition, by screening large numbers of cells at decreased resolution we can define the percentage at which different cell types are present in our preparation. With the second example, the root tip of cress, we illustrate how combining information from intermediate resolution data with high resolution data from selected regions of interest can drastically reduce the amount of data that has to be recorded. By imaging only the interesting parts of a sample considerably less data need to be stored, handled and eventually analysed.

**Conclusions:**

Our custom-designed substrate holder allows reproducible generation of section libraries, which can then be imaged in a hierarchical way. We demonstrate, that EM volume data at different levels of resolution can yield comprehensive information, including statistics, morphology and organization of cells and tissue. We predict, that hierarchical imaging will be a first step in tackling the big data issue inevitably connected with volume EM.

**Electronic supplementary material:**

The online version of this article (doi:10.1186/s12860-016-0122-8) contains supplementary material, which is available to authorized users.

## Background

In view of the recent success of super resolved fluorescence light microscopy or nanoscopy, as it is also called by one of the Nobel awardees [1], the question arises how relevant electron microscopy (EM) will be for the future of the life sciences. When it was introduced not quite 100 years ago it was not exactly a method suited to image entire cells or even complete model organisms at nanoscale resolution. However, new developments in volume EM [2, 3] are challenging that statement.

There are several ways to create volume EM data: The blockface methods, serial blockface scanning electron microscopy (SBFSEM: [4]) and focussed ion beam scanning electron microscopy (FIBSEM, reviewed in [5]), are well established in the field. Here the surface or blockface of a sample embedded in a resin block, is alternately imaged and removed in a cyclical manner in a SEM. Both methods are destructive, consuming the sample while it is being imaged. For SBFSEM this can lead to the necessity of imaging very large areas, in extreme cases the whole blockface, at rather high resolution, because it is not possible to rescan interesting areas later. In this way huge data sets (cf. [2]) are produced which may contain only few regions with really interesting events or substructures.

Another possibility to explore the third dimension with EM is the array tomography (AT) approach where arrays of ultrathin serial sections are deposited on large, solid substrates and imaged either in a light microscope (LM) or in a SEM. The method was originally introduced for multiplexing immuno-staining by repeated stripping and re-labelling of the section arrays in order to map synaptic connections in brain [6, 7]. In the neurosciences field, that pioneered all volume EM techniques (reviewed in [8]), variations of the original method are quite common, also extending it to SEM imaging (reviewed in [2, 9]). However, applications in cell and developmental or even general biology are rather scarce up to now [10–12]. One advantage of this method is its potential for hierarchical, targeted imaging, which we will illustrate with examples in the present paper. AT also allows correlative or conjugate [13] approaches, when substrates amenable to LM are used. To this end we developed a tool that helps to reliably create arrays of sections on a number of different substrates, suitable for SEM as well as for different modalities in LM.

## Results

### Custom-built substrate holder as prerequisite for reliable retrieval of multiple ribbons

**Fig. 1:**
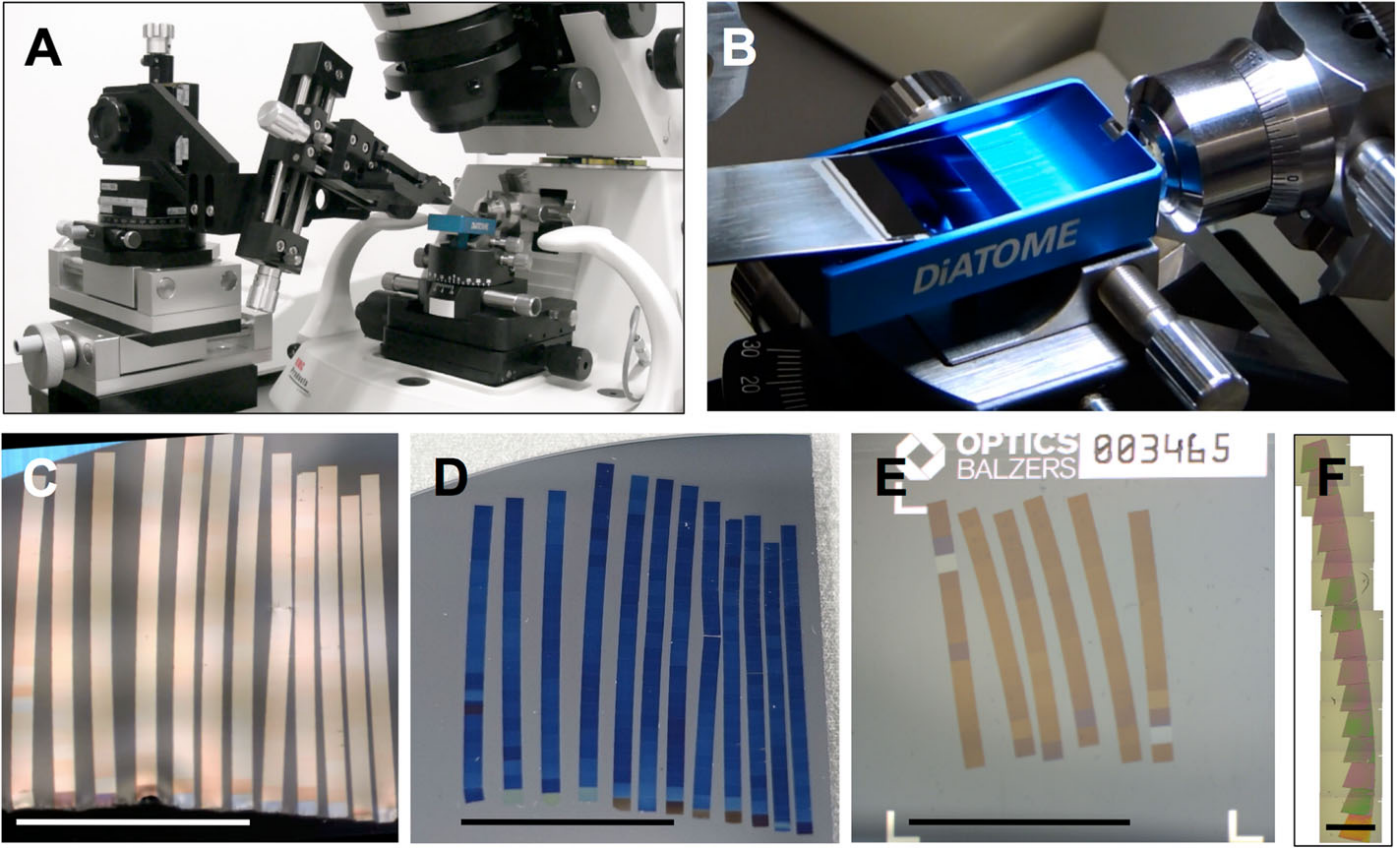
Creating arrays of several ribbons with a custom-built substrate holder. Ultramicrotome with substrate holder attached (**a**), jumbo diamond knife with large boat that can harbour substrates up to microscope slide size (**b**). 12 ribbons (about 270 sections total) attached to substrate, still floating on water surface in knife boat (**c**) and the same sections dried onto a piece of silicon wafer after lift-out (**d**). For correlative light and electron microscopy ITO-coated, transparent coverslips (**e**) are the substrate of choice. Section thickness can range from ultrathin (**c**-**e**) to semi-thin (**f**). Scale bars: 10 mm in C, D, E, 1 mm in F

The dominating problem when cutting serial sections is the successful retrieval of the sections from the knife boat in an ordered manner. To overcome this, the ATUMtome has been developed [14] which automatically collects thousands of serial sections on a plastic tape as individual, separate entities. The main disadvantage of this device is the fact, that the tape is not suitable for advanced light microscopy techniques, such as super resolution LM. In addition, contrary to connectomics, where indeed huge volumes need to be processed, questions in cell or developmental biology often ask for “only” several hundred sections. Having started to collect ribbons of sections manually onto solid substrates more or less successfully, we analysed all movements of the operator while doing this and came up with a new device (Fig. 1a, see also Additional file 1: Figure S1, Additional file 2: Figure S2, Additional file 3: Figure S3) allowing 7 degrees of free movement. The governing principle in the design of the substrate holder is the selection and arrangement of translation and rotation axes that on the one hand allow precise positioning and manipulation of the substrate (axes #4-#7) and on the other hand convenient adaptation and operation on different microtomes (axes #1-#3) and tables. Moreover besides adjustment of axes #1-#3, the holder can be reconfigured for operation on different microtome types and tables by changing the base holder support and the traverse. Depending on the type and supplier of the microtome the position where the knife is sitting on the microtome varies. Furthermore the distance of the knife to the front side of the table changes, depending on the position of the microtome on the table. Both parameters, the distance of the knife to the table front side and the height of the knife above the table top, may change when adapting the holder to a new lab situation. Apart from these, depending on the microtome type, the contour and the dimensions of the microtome main body have to be taken into account as well when fitting the substrate holder to the microtome. We change the traverse to adapt the offset between holder base and substrate clamp and the base holder support to adapt to knife height. Our holder has been adapted to, tested and used with a standard TMC vibration isolation table and after adaption of the base table fixation clamps with a custom-built Accurion table with integrated Halcyonics i4 active vibration isolation platform. Moreover we adapted to and applied it on two different ultra microtomes while developing our process (RMC Powertome, Leica UC7). In every single of these different configurations the substrate holder enabled the user to reliably deposit several long ribbons of serial sections onto one substrate (Fig. 1d, e). To be able to fit substrates up to the size of a conventional glass slide for LM into the knife boat, a Jumbo knife (Fig. 1b) has to be used. The actual substrate, e.g., a piece of silicon wafer (Fig. 1d) or a special glass coverslip (Fig. 1e) coated with indium tin oxide (ITO) is attached to a slide-sized supporter with a peelable adhesive and inserted into the knife boat (Fig. 1b and also Additional file 4: Movie S1). Ribbons of sections are directed away from the knife’s edge to the place where the water touches the substrate and attached to the dry part of the substrate that sticks out of the water (cf. Fig. 1c). Having collected a number of ribbons in that way, the substrate is smoothly lifted out of the water using the micropositioning stages (see also Additional file 5: Figure S4 for different lift-up trajectories). The movement can be controlled in such a way that even on substrates with rougher surfaces (e.g., ITO) no rupturing or other disturbance of the ribbons is observed. Besides standard ultrathin sections with a thickness ranging from 50 to 100 nm, ribbons consisting of semi-thin sections up to 1 μm thickness (Fig. 1f) have been handled.
**Additional file 4: Movie S1** Substrate holder in action. (MP4 16804 kb)

### Multi-scale imaging of large volumes on arrays of ultrathin serial resin sections

**Fig. 2:**
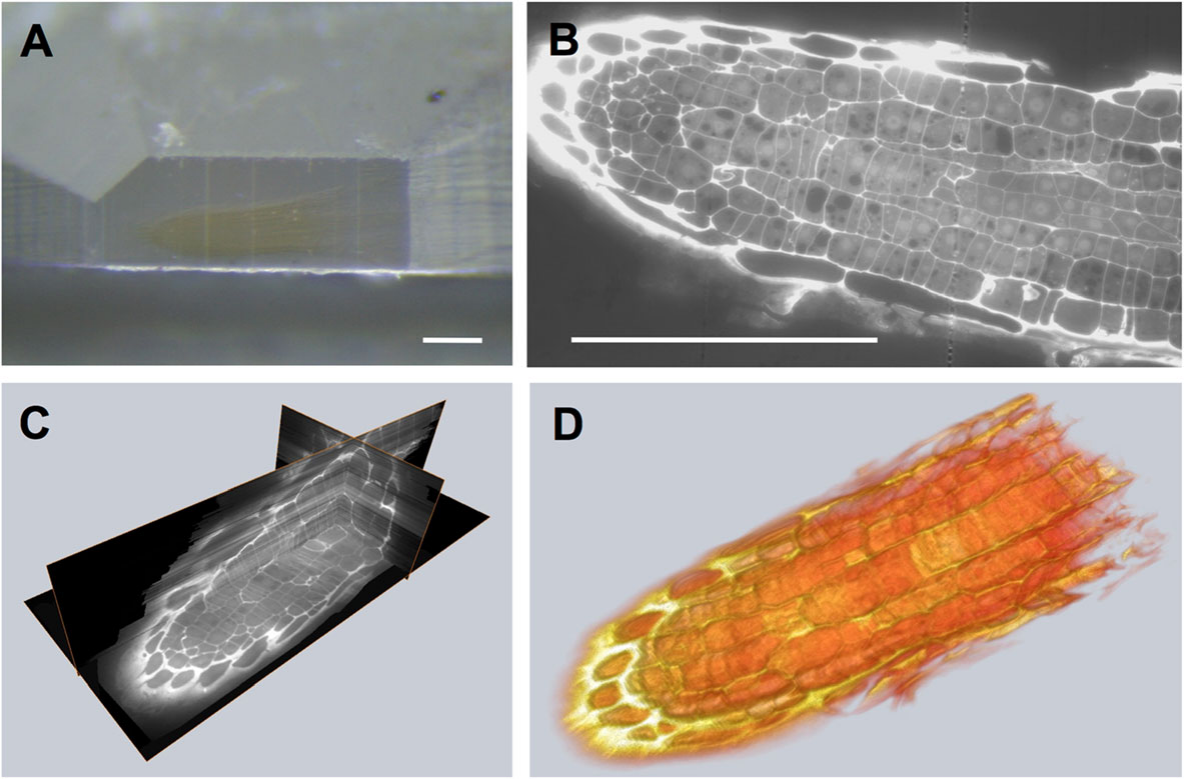
Fluorescence light microscopy on serial sections from Arabidopsis roots. Arrays produced from Arabidopsis roots embedded in HM20 (**a**) were stained with propidium iodide and imaged in a standard wide field fluorescence microscope (**b**). 3D reconstructions from a stack of 200 sections were visualized in Amira as orthoslices (**c**) or by volume rendering (**d**). Scale bars: 100 μm

As shown in the initial paper on AT [6], the method is well suited for imaging with both photons and electrons. When mounting sections derived from samples embedded in hydrophilic resin, e.g., Lowicryl HM20 (Fig. 2a) on transparent substrates, such arrays may be labelled with antibodies or stained with water-soluble dyes, here propidium iodide, and imaged in a standard wide-field fluorescence microscope (Fig. 2b) with rather good z-resolution. Since the section thickness of 100 nm is thinner than the z-discrimination of a confocal microscope, even highly over-stained samples can still deliver excellent images. A reconstruction from 200 sections, aligned in TrakEM [15] is visualized as orthoslice (Fig. 2c) or volume rendering (Fig. 2d). This represents about 20 μm or about 1/6 of a complete root tip’s width.

Imaging with electrons relies on heavy metals to deliver good signals, so we used samples as typically prepared for transmission electron microscopy. During the preparation, osmium and uranium were incorporated into the block and the sections were additionally post-stained with uranium and lead. Initial manual imaging of arrays in the SEM proved very tedious, recording 70–100 sections for the 3D reconstruction of a small cell easily required several hours of rather concentrated work. More recently, we were able to test as early adopters the newly released ZEISS Atlas 5 Array Tomography platform (Carl Zeiss Microscopy, GmbH, Oberkochen, Germany). The platform consists of a scan generator that can create images of up to 32k x 32k, which in turn may be stitched together to even larger “regions” using the software package supplied with it. The software takes over the command of the microscope and can also be used to image every section of the array (section sets) and subsequently predefined regions of interest (ROIs) within every section (site sets). This solution allows hierarchical imaging at different resolution levels in an automated manner, as explained below.

**Fig. 3:**
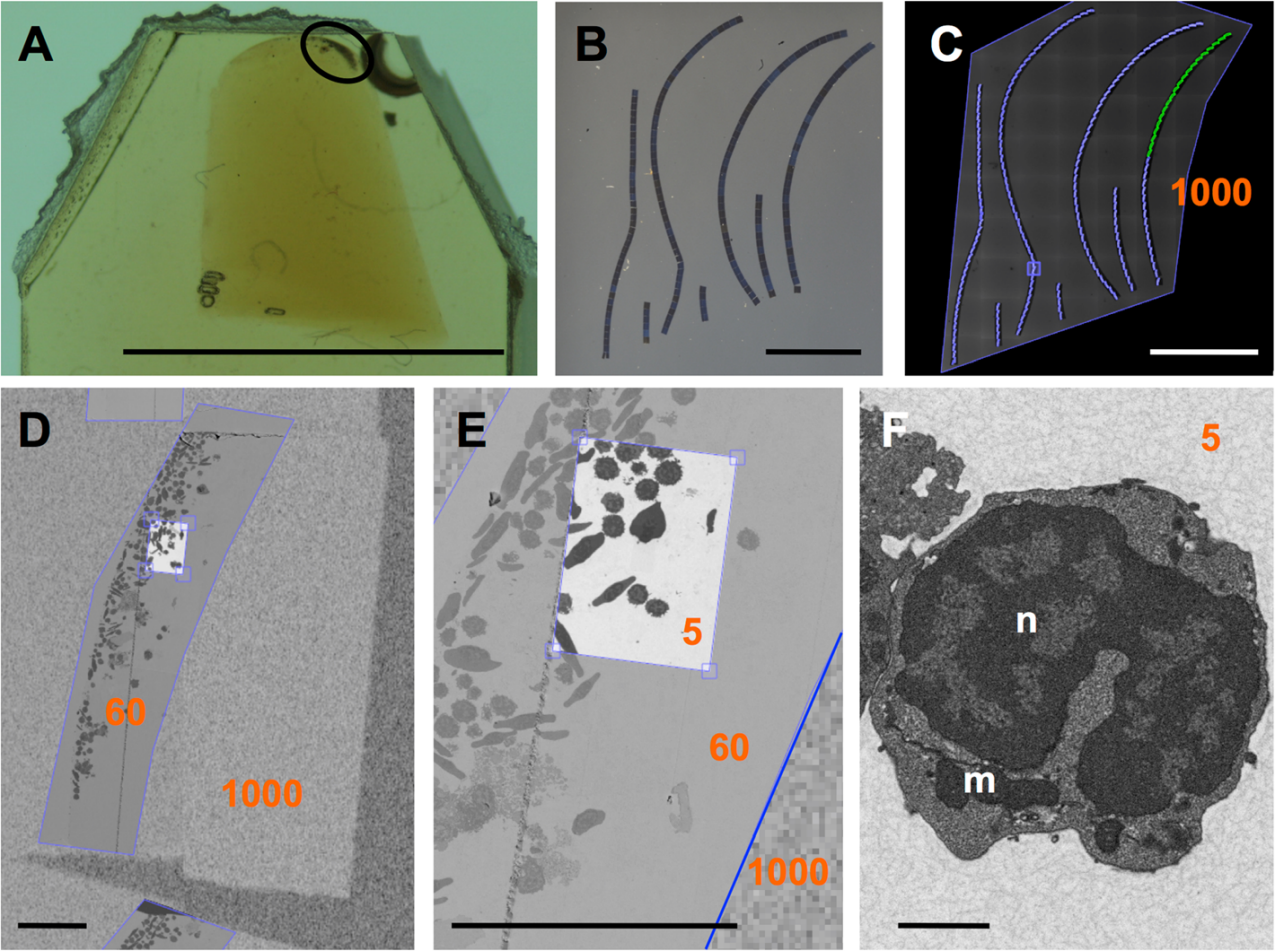
Hierarchical imaging. Arrays of several ribbons are sectioned from resin blocks (**a**) here containing a pellet of zebrafish immune cells (circle) and deposited on silicon wafers (**b**). Overview of array imaged in a FESEM with 1000 nm pixel size (**c**), whole pellet in one section imaged with 60 nm pixel size (**d**), ROI imaged with 5 nm pixel size (**e**), and zooming in to one cell in this ROI (**f**). Numbers in orange indicate pixel size in the respective ROI. Note the clearly visible change in pixel size at the borders (blue frames) of the ROIs in D, E. Scale bars: 5 mm in A-C, 50 μm in D, E, 1 μm in F; *n* = nucleus, m = mitochondria

Our first example is a pellet of zebrafish (*Danio rerio*) blood cells isolated from the lymphoid gate of spleen by FACS and first enrobed in an agarose plug (conical shape in Fig. 3a) before being prepared as for classical EM. An array of about 200 sections was imaged with a digital camera (Fig. 2b) and then in a FESEM (field emission scanning electron microscope) with 1000 nm pixel size (Fig. 3c, a “region” consisting of about 70 stitched tiles), which allowed identification of the outline of the cell pellet in the individual sections. Next an irregularly shaped ROI enclosing just the cell pellet was used for setting up a section set (Fig. 3d, blue outlines). This was imaged with a 60 nm pixel size (Fig. 3d) and used to search for interesting cells and also to define the number of sections necessary to enclose an entire cell in z-direction. On these sections a second, smaller ROI (site set) was placed at the appropriate positions and imaged at a 5 nm pixel size (Fig. 3e, inner box). This resolution is sufficient to define membrane-bound compartments within a cell, shown here are nucleus, mitochondria, and ER (Fig. 3f). It is interesting to note the different pixel sizes at the transitions from one ROI to the next, e.g., in Fig. 3d and e. With the recording software it is also possible to navigate and zoom seamlessly through the recorded image data from the macro-scale (the whole array is about 1 cm wide) to the nanoscale, as illustrated in Additional file 6: Movie S2.
**Additional file 6: Movie S2** Hierarchical imaging on an array of 200 serial sections. (MOV 18307 kb)

### Nanomorphomics in a cell population – organelle inventories and more

**Fig. 4:**
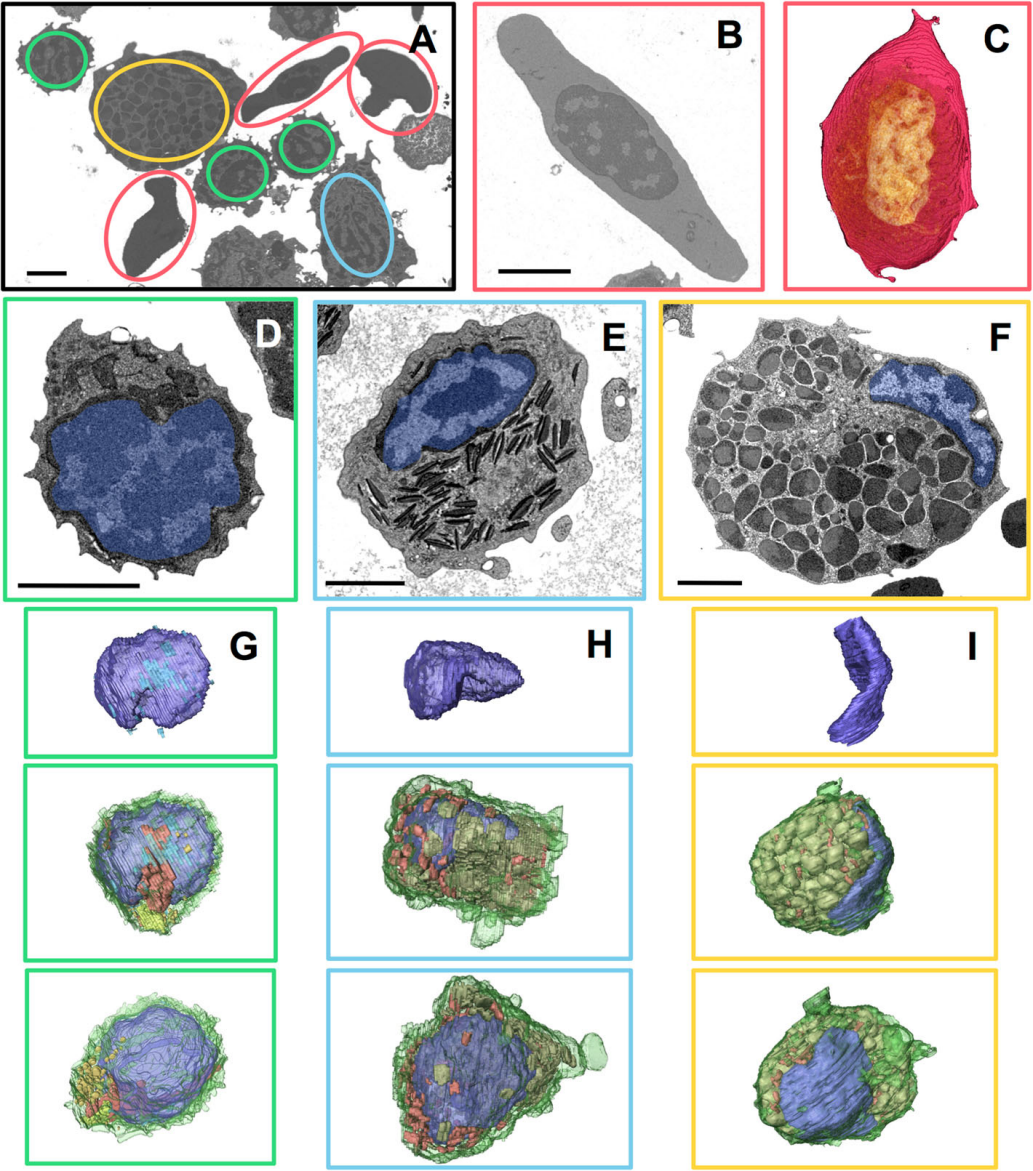
Organelle inventories of different cells in a zebrafish immune cell population isolated from spleen. Overview of ROI imaged with 5 nm pixel size (**a**), containing RBCs (*red*, **b**, **c**), cytotoxic cells (*green*, **d**, **g**), neutrophils (*cyan*, **e**, **h**) and basophils (*yellow*, **f**, **i**). Representative cross sections from 3D data sets of 40–100 sections of these cells (**b**, **d**-**f**), a volume rendering for an RBC (**c**) and segmented 3D data (**g**, **h**, **i**) showing nucleus in dark blue, ER in light blue, mitochondria in red, Golgi apparatus and secretory lysosomes in yellow, neutrophilic granules in brown, basophilic granules in gold, and plasmamembrane in green. Scale bars, 2 μm

From high-resolution image stacks containing different cell types (representative slice shown in Fig. 4a), individual cells can be reconstructed and volume rendered, such as a red blood cell (RBC) (Fig. 4b and c), which in fish display mainly nucleus and plasma membrane. When more organelles are present, segmentation is usually necessary to define the various organelles. Shown here are a cytotoxic cell (Fig. 4d and g, Additional file 7: Movie S3) representing the largest subpopulation (cf. Table 1), a neutrophilic granulocyte (Fig. 4e and h, Additional file 8: Movie S4, Additional file 9: Movie S5), and a basophilic granulocyte (Fig. 4f and i, Additional file 10: Movie S6). They have similar basic organelle inventories – nucleus, mitochondria, Golgi, ER, but different sets of granules (Fig. 4g-i). Considering their primary function this is not an unexpected finding. However, morphology and positioning of the nuclei vary considerably too, as does the volume ratio of nucleus to cytoplasm. In the cytotoxic cell there is very little space between nuclear envelope and plasma membrane, except on one side of the nucleus where most organelles are concentrated in a dip of the nucleus (middle image in Fig. 4g), creating a highly asymmetric cell. The neutrophilic granulocyte has more cytoplasm, its nucleus is usually also lobed and displaced to one side of the cell with the bulk of cigar-shaped granules and other organelles on the opposite side (see also Additional file 7: Movie S3, Additional file 8: Movie S4 for a more detailed view). For the basophilic granulocyte the distribution is even more extreme, the nucleus is flattened against one side of the cell while the globular granules take up most of the space.
**Additional file 7: Movie S3** 3D representation of cytotoxic immune cell from zebrafish spleen. (MOV 5255 kb)

**Additional file 8: Movie S4** Image stack of complete neutrophilic granulocyte with segmentation. (MOV 1744 kb)

**Additional file 9: Movie S5** 3D representation of neutrophilic granulocyte from zebrafish spleen. (MOV 5178 kb)

**Additional file 10: Movie S6** 3D representation of basophilic granulocyte from zebrafish spleen (MOV 4839 kb)

**Table 1:** Different cell types in a FAC-sorted population from zebrafish spleen

Cell type	n	%
RBC	88	36.8
cytotoxic cell	114	47.7
neutrophil	4	1.7
basophil	3	1.3
others	30	12.5

Apart from studying cell morphology at nanoscale resolution, arrays can also be used for quantification and statistics, an attribute not commonly associated with EM. The FAC-sorted population from the lymphoid gate of spleen investigated here was sorted with a rather wide gate to increase yield. The pellet shown in Fig. 3a consisted of about 50,000 cells, the yield from the spleens of five adult fish. Analysis of such a small pellet at that nanoscale resolution would be very difficult by other means. The different cell types can be counted, theoretically in the whole pellet if one would cut it up completely. In practice, sufficiently high numbers of cells can be counted by choosing individual sections far enough apart in z to exclude counting a profile from the same cell twice. For identification of a cell type, intermediate resolution (here a series imaged with 60 nm pixel size) is sufficient: Once a whole cell of a given type has been reconstructed in 3D at high resolution, characteristic features will help to identify the cell type in a single section at lower resolution. Examples are the prominent granules of the granulocytes or the characteristic cogwheel shape of the cytotoxic cell. In this manner 239 cells were counted on three different sections (cf. Table 1).

### Tackling tissue – polarity in a root tip

**Fig. 5:**
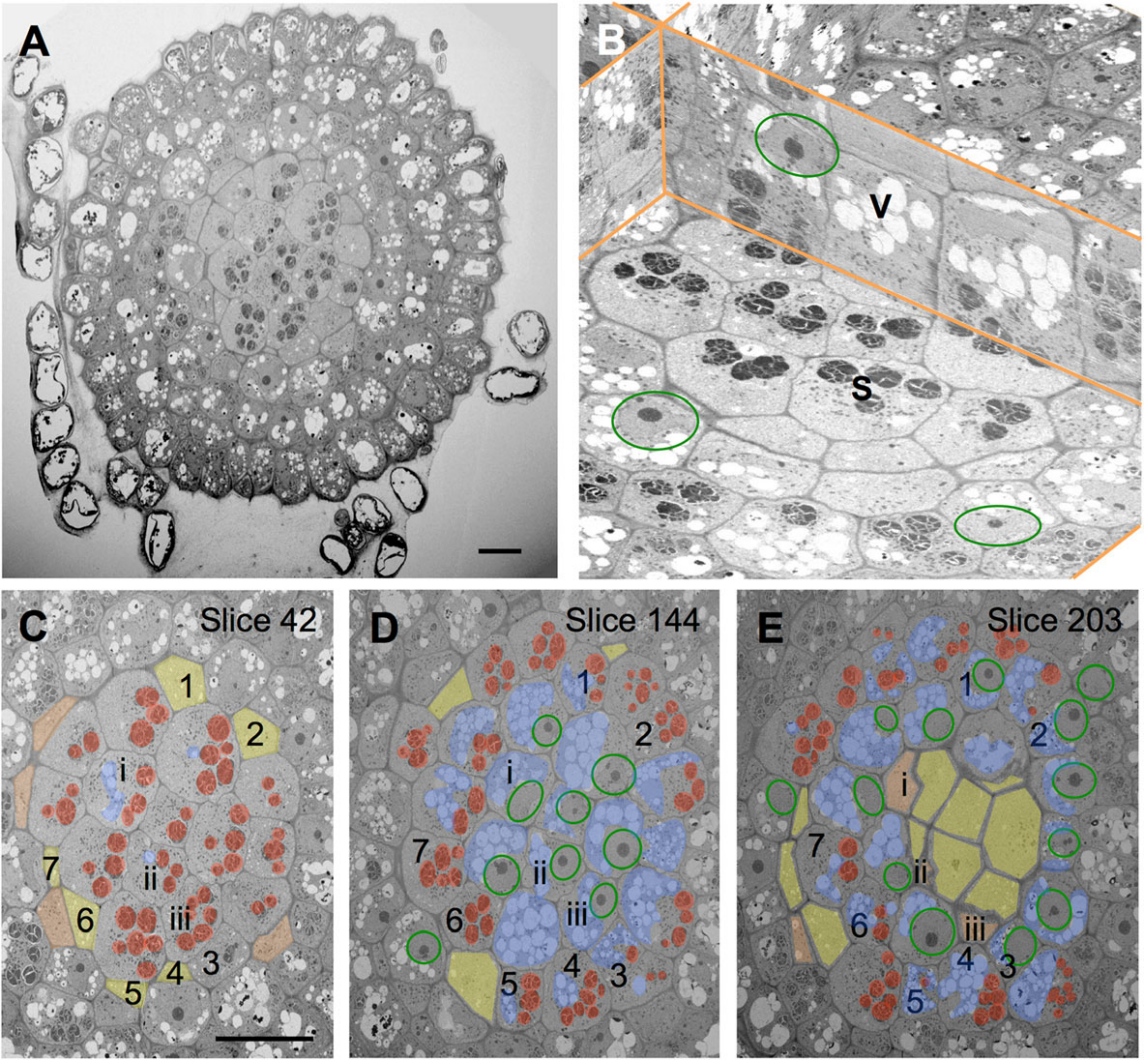
Tissue organisation in the central column of the root calyptra. In a volume of 200 × 200 × 24 mm reconstructed from cross sections (representative example in **a**) across a whole cress root the centre (orthogonal views in **b**) shows distinctly polar distributions of the larger organelles, such as statoliths (S, *red*), vacuoles (V, *blue*), and nucleus (*green* circles). In particular slices (**c**-**e**), cohorts of cells (1–7 and i-iii) contain comparable organelle sets. Cell profiles coloured yellow represent the apical part, orange the basal part of the respective cell. Scale bars: 20 μm

For the 3D reconstructions of single cells shown so far, relatively small numbers of sections, between 50 and 100, were sufficient to cover the whole cell. This number will soar when we begin to look at tissue. We illustrate that with an example from the plant world, the root calyptra from cress (*Lepidium sativum*). In 240 cross sections (each 100 nm thick), starting about 20 μm from the tip we recorded the whole root profile at 60 nm pixel size (Fig. 5a). This allows recognizing the large organelles in the 3D reconstruction (Fig. 5b), such as big vacuoles, statoliths (starch grains functioning as gravity sensors) and the nucleus with its prominent nucleolus. Looking at the volume in xz-direction it becomes obvious that these organelles are not randomly distributed within a cell, but are found in distinct zones. Looking at cross sections on different levels in the volume (Fig. 5c-e) it is apparent that groups of cells are forming cohorts, with their profiles exhibiting similar organelle contents at the same level along the root axis. For example the profiles of the cells numbered 1–7 (Fig. 5c-e) are devoid of any larger organelles in slice 42 (Fig. 5c), contain only statoliths or statoliths and vacuoles in slice 144 (Fig. 5d), and statoliths and vacuoles or vacuoles and nuclei in slice 203 (Fig. 5e). On the other hand, cells i-iii, with only statoliths in slice 42 and nuclei and vacuoles in slice 144 have almost disappeared in slice 203, indicating a defined layering of organelles along the root axis.

**Fig. 6:**
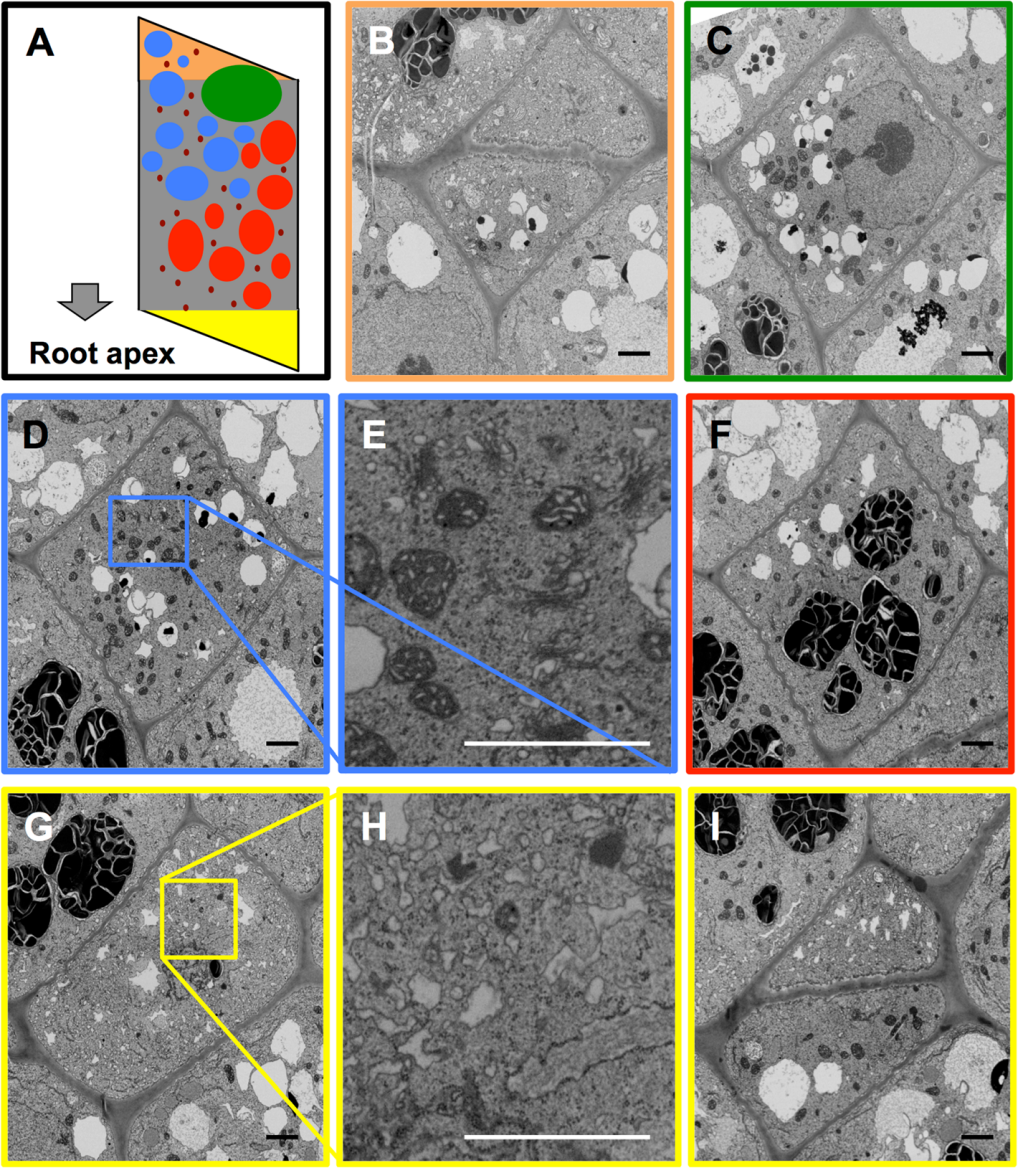
Polarization of calyptra cells. Individual slices from a stack recorded with 5 nm pixel size. The coloured frames around the images **b**, **c**, **d**, **f**, **g**, **i** indicate in which zone the slice is located, compare also with scheme (**a**). The frames in **d** and **g** are shown enlarged in **e** and **h** respectively. Colour codes are red for statoliths, blue for large vacuoles, green for nucleus, auburn for mitochondria, yellow for the apical zone without any large organelles, and orange for the distal zone. Scale bars: 5 μm

To show the polar distribution of the larger organelles and to find out whether smaller organelles, such as mitochondria, dictysomes and other compartments of the secretory pathway exhibit a similar arrangement we recorded an individual cell with 5 nm pixel size, allowing identification of all organelles down to the size of ribosomes. Representative cell profiles (Fig. 6b-i) moving from distal (Fig. 6b) to apical (Fig. 6i) indeed show that there are distinct zones within the cell parallel to the longitudinal axis of the root. The smaller organelles e.g., mitochondria and dictyosomes (Fig. 6e), are distributed throughout the cell, with the exception of the zone close to the apical cell wall. This zone is devoid of any larger organelles and contains only an extended and convoluted system of membranes (Fig. 6h). These findings are summarized in a scheme (Fig. 6a).

Here we would like to point out an emerging and serious problem with big data: Imaging at high resolution implies that new ways of displaying data are needed. The cell presented here is so big that it is not possible to display the distribution of large and small organelles in the same image when viewing the whole cell as in Fig. 6. This can be alleviated by combining overviews and close-up images at several resolutions in movies (Additional file 11: Movie S7, Additional file 12: Movie S8, Additional file 13: Movie S9) presenting more detailed views of selected slices within the total volume. However, modern 3D virtual reality displays and walks through reconstructed structures will certainly be necessary to fully understand the entire nanoscale organisation of complex cells and tissue.
**Additional file 11: Movie S7** Zooming in to slice 36 of a cell in the cress root calyptra. (MOV 17394 kb)

**Additional file 12: Movie S8** Zooming in to slice 159 of a cell in the cress root calyptra. (MOV 11655 kb)

**Additional file 13: Movie S9** Zooming in to slice 227 of a cell in the cress root calyptra. (MOV 11852 kb)


## Discussion

Based on the AT approach introduced in 2007 [6] we propose an easy access workflow for multi-scale hierarchical imaging applicable not only to model organisms with their dedicated genetic tools, but to many types of samples, even unique ones. Some preliminary, technical details of this workflow were already presented as abstracts to a specialized microscopy audience [16, 17].

Our custom-built substrate holder is a relatively low cost attachment – which can be retrofit - to a common ultramicrotome, an instrument available in virtually every EM lab or facility. One of the first substrate holder devices was introduced in 1964 by Behnke & Rostgaard [18], consisting of a stand with a cantilever and a clamp mounted on the free end of that cantilever. The clamp can hold a pair of forceps, which in turn holds the TEM grid. A rack and pinion drive between cantilever and clamp allows moving the substrate longitudinally. Very similar devices have been presented over the years [19, 20]. With only one or two degrees of freedom the adaptability to fit an ultramicrotome setup with a rotated knife is insufficient. This is the main disadvantage of these devices. To overcome this limitation Meyer & Domanico [21] introduced a device that is attached directly to the knife or the knife holder. This device has always the same orientation as the knife. It supports one TEM grid and the lift out movement can be motorized. Because of its focus on TEM grids it is not intended to use other substrates. Other supporting devices for TEM grids not needing any mechanical parts are also described: One idea is a modified knife boat to hold the grid under the water [22]. It has also been described how to attach the grid on the floor of the knife boat next to the knife edge [23]. All these modifications are limited to TEM grids, too.

The latest device published is designed for cryo-ultramicrotomy [24]. It consists of two micromanipulators, each offering a three-way movement. One micromanipulator holds the forceps gripping the TEM grid, the other the conducting fibre for manipulating the sections. This device is designed only for one specific microtome and does not offer the adaptability mentioned before.

With our device we have an element of freedom in planning our experiment since we have a slide-sized carrier onto which we can mount a wide variety of different substrates – in principle from TEM grids to glass coverslips or silicon wafers. We also have high flexibility for sample orientation within the block since the substrate holder can be aligned with a rotated knife, which was not possible for any of the previous devices.

The ATUMtome [14, 25], an automated sectioning device using a carbon-coated Kapton tape to pick up sections, is a rather complex device with constraints reducing the field of application. It does not have e.g., the option to use a glass substrate, which will facilitate super resolution LM [26] and also correlative imaging [27]. With the ATUMtome it is difficult to collect thick (1–5 μm) sections e.g., for histology, because ultrathin sections adhere much better and the rolling of the tape may lead to loss of thick sections. With our device it is no problem to produce and pick up ribbons of 1 μm thick sections.

The possibility to section physically allows very high z-discrimination in a standard wide-field fluorescence microscope down to 50–70 nm, depending on how thin the sections can be made.

Since post-staining of arrays can be done with exactly the same reagents as used in traditional TEM imaging, high metallization of samples, which is necessary for the blockface methods SBFSEM and FIBSEM, is not required. We were also able to successfully apply this workflow to human pathology samples prepared according to standard protocols with osmium as the only heavy metal in the block (not shown).

Manual imaging of arrays in the SEM at low voltage is possible, but very time consuming. Here automated imaging, specifically in combination with hierarchical imaging is a decided advantage. Contrary to the blockface imaging methods (SBFSEM and FIBSEM nanotomography), where just one imaging cycle is possible, AT allows revisiting ROIs and imaging them at different resolutions. Precious or unique samples are preserved, albeit in a “sliced-up” version. This is not the case with SBFSEM and FIB nanotomography where the samples are consumed by the process.

In addition, targeting specific structures within a tissue or rare events in a population of cells, such as e.g., identifying immunological synapses in a coculture of cytotoxic cells and cancer cells [10] can be realized rather easily on arrays. Medium resolution images are sufficient to screen whole pellets for the desired events. Then ROIs are only placed on the sections displaying these and imaged at the resolution required to analyze the corresponding structures in detail. For the blockface methods, much more complicated procedures such as laser branding and CLEM (correlative light and electron microscopy) are necessary to target the volume of interest in the whole block [28].

One disadvantage of AT for EM is founded in the imaging properties of the SEM. Since we can only image the surface of a section, resolution in z-direction is limited to the section thickness, leading to discrete sampling of the volume and often highly anisotropic voxels (see also Table 2). For most examples shown here this was not a serious limitation – organelle inventories or distributions of organelles along the axis of an organ can still be established, even with sections as thick as 100 nm.

**Table 2:** Dimensions of volumes, data, and acquisition time

sample	image size (μm)	# of sections	voxel (nm)	data (GB)	imaging time/frame	imaging time total
spleen - whole pellet	387 × 163	180	60 × 60 × 100	1.4	2 min^a^	2 h
spleen - several cells	30 × 22	108	5 × 5 × 100	3.3	10 min	18 h
root - cross section	246 × 246	242	60 × 60 × 100	4.1	7 min	28 h
root - one cell	30 × 28	242	5 × 5 × 100	8.1	13 min	52 h

^a^SE detector used, 6.4 microseconds dwell time

If higher resolution in z is required, FIBSEM nanotomography on selected sections is an option we are currently exploring [29].

Another point illustrated by our range of examples is the relationship between physical volume size, resolution, data size and imaging time (Table 2). For example imaging of a whole root cross section (ca 250 × 250 μm) over 24 μm (240 sections) with 60 nm pixel size took 28 h and produced 4.1 GB of data. At this resolution only the larger organelles within the cells were visible. Increasing resolution to 5 nm pixel size to allow detection of all membrane-bound organelles and ribosomes increased imaging time for just one cell (ca 30 × 30 μm image size, again 240 sections) to 52 h with 8.1 GB of data. Microscope settings were comparable for both datasets (cf. Methods section). To image all of the 30 cells in the central column would have taken 65 days, if that would have been productive is another question. The combination of information obtained from the intermediate resolution data with information obtained from a representative volume imaged at high resolution was already sufficient to extract enough facts for building a tentative model of those cells’ polar organization.

## Conclusions

To ensure reliable retrieval of ribbons of serial sections for AT from the boat of a diamond knife we introduce a substrate holder with 7 degrees of free movement specifically designed for that purpose. Using this we are able to deposit up to two hundred sections densely packed in an ordered way on an area the size of a 22 × 22 mm coverslip. When creating such arrays on substrates amenable to LM they can be imaged with very good z-discrimination even in an ordinary wide-field fluorescence microscope. Arrays on silicon wafers were imaged in a hierarchical way in a SEM using a software platform for automated imaging. Hierarchical imaging is an easy way to target rare events or substructures within a larger context. Adapting image collection in the SEM resolution-wise to the question being investigated can help to reduce the amount of data produced. Finally, combining both imaging modalities opens the way to large volume correlative approaches.

## Methods

### Sample preparation

Arabidopsis roots were high pressure frozen, freeze substituted and embedded in Lowicryl HM20 as described [30].

Immune cells were isolated from the spleens of five adult zebrafish, chemically fixed and embedded in epoxide resin as described previously [10].

From cress seeds germinated for 3–4 days on wet filter paper the roots were cut off and immersed in 1% glutaraldehyde in 50 mM cacodylate buffer at 4 °C over night. After 4× 10 min washing in buffer they were postfixed in 1% OsO_4_ in cacodylate for 4 h at room temperature, followed by further washes, 2× 10 min in buffer and 2× 10 min in double-distilled water, they were block-stained over night at 4 °C with 1% uranyl acetate in double-distilled water. Next steps were: Further washing, 4× 10 min in double-distilled water; dehydration in a graded acetone series of 25%, 50%, 75%, and 2× 100% for 15 min each; infiltration in Spurr’s resin for 45 min each in 25%, 50%, 75% resin and over night in 100% resin at 4 °C. Before embbeding in fresh resin in BEEM capsules, 100% resin was exchanged once and kept for several hours. Resin was polymerized at 60 °C for 1 d.

### Concept of the custom-built substrate holder

The holder is based on the supporting hand concept. It allows the operator to position the substrate in the boat accurately while optimising the waterline between water and substrate, which depends on the substrate material used and the contact angle. After positioning of the substrate the operator gains one hand free for other purposes. The substrate can be positioned in a wide range and even a knife rotation around the vertical axis of up to +/− 10° can be handled. To meet the +/− 10° knife rotation a lateral coarse positioning of the holder base with a travel range of +/− 25 mm has been integrated (coarse translation axes #1 and #2, see also Additional file 2: Figuer S2). The rotation of the substrate clamping unit around the vertical axis can be realised using rotation axis #3. Axis #3 allows an endless turning and is also used to rotate the holder mechanism out of the knife work space e.g., when changing the knife for trimming. The substrate position in the knife boat can be changed using axis #4 for off-centre movement (side-ways) and axis #5 for vertical positioning of the substrate (longitudinal positioning of the waterline). The substrate water surface angle can be set with axis #6 and finally the longitudinal movement of the substrate towards the knife is realised with axis #7. The lifting process after pinning all ribbons or sections at the substrate is realised using axes #5 (vertical lifting) and #6 (substrate rotation towards horizontal, see also Additional file 5: Figure S4).

Additionally to the translation and rotation stages used for axes #1-#7 offering the described movements we integrated several passive adjustment possibilities based on slotted holes allowing e.g., the adaptation of the position of the substrate rotation angle axis in relation to the substrate. Furthermore slotted holes can be used to adjust the substrate clamp to shorter substrates, the fine-tuning of the height of the substrate holder, and the middle position of axis #6. Table 3 gives an overview of the axes used in the holder.

**Table 3:** Axis travel ranges

Axis #	Axis name	Travel range	Comment
#1	Base coarse positioning 1	+/− 25 mm	perpendicular to table front side
#2	Base coarse positioning 2	+/− 25 mm	parallel to table front side
#3	Substrate vertical rotation	endless	large offset from substrate position
#4	Substrate off-center positioning	+/− 10 mm	side-ways along knife edge direction
#5	Substrate vertical positioning	+/− 10 mm	Substrate lowering and lift off movement
#6	Substrate angular rotation	+/− 20°	angle between water surface and substrate
#7	Substrate longitudinal positioning	+/− 25 mm	in substrate plane towards/away from knife edge

### Producing arrays of sections

Polymerized resin blocks were trimmed and the leading and trailing edges of the blockface coated with a mixture of 30% glue (Pattex, Henkel; Germany) in xylene to stabilize the section ribbons. Serial sections, usually 70 nm to 100 nm thick, were cut either on a UC7 ultramicrotome (Leica, Germany) or a Powertome PC (RMC Boeckeler, USA) using a Jumbo knife (Diatome, Switzerland). A custom built handling device helped to place several ribbons on small pieces of silicon wafers or ITO-coated glass coverslips (Optic Balzers, Liechtenstein). Arrays produced from methacrylate resin were stained with 1 μg/ml propidium iodide (Sigma-Aldrich, USA) in distilled water over night at 4 °C. Arrays from epoxide resin were post stained with uranyl acetate and lead citrate as described before [10].

### Recording image data

Fluorescence images were recorded in a Cell Observer (Carl Zeiss Microscopy GmbH, Germany) wide-field fluorescence microscope equipped with a mercury arc lamp (Illuminator HXP 120 V) and a rhodamine filterset.

Imaging of arrays with electrons was done in a Crossbeam 540 (Carl Zeiss Microscopy GmbH, Germany), a field emission SEM featuring a double condensor system. Imaging conditions were: 1.5 kV, a beam current of 811 pA, ESB (energy-selective backscatter) detector, grid at 1000 V, 25.2 microseconds dwell time. Using computer-assisted tools in the newly released ZEISS Atlas 5 Array Tomography platform, serial sections were imaged at multiple resolutions: First the whole array (“region”, mosaic of about 70 tiles) was imaged automatically at 1000 nm image pixel size. Then serial sections were recorded automatically at 60 nm pixel size (“section set”). On these section images interesting cells or groups of cells were selected for further high-resolution imaging (“site sets”). These ROIs were automatically imaged over a range of 50–250 serial sections at 5 nm pixel size using a large single (up to 32 k × 32 k pixels) frame for each site.

### Image processing

Image stacks recorded with ZEISS Axiovision (Carl Zeiss Microscopy GmbH, Germany) or exported from ZEISS Atlas 5 Array Tomography were cropped and registered using the stackreg plugin [31] or TrakEM2 [15] in Fiji [32].

Subsequent volume rendering and segmentation were performed with the Amira software package (VSG/FEI, USA). Movies were produced in ZEISS Atlas 5 (Google Earth-like) or Amira (segmented or rendered volumes).

## Additional files

Additional file 1: Figure S1. 3D CAD model of substrate holder, side view right side (TIFF 3603 kb)
Additional file 2: Figure S2. 3D CAD model of substrate holder, top view – 10° knife rotation (TIFF 3524 kb)
Additional file 3: Figure S3. 3D CAD model of substrate holder, front view (TIFF 3032 kb)
Additional file 5: Figure S4. Substrate lift-up trajectories (TIFF 915 kb)

## Abbreviations

AT: Array tomography
EM: Electron microscopy
FACS: Fluorescence activated cell sorting
FE: Field emission
FIB: Focused ion beam
ITO: Indium tin oxide
LM: Light microscopy
RBC: Red blood cell
ROI: Region of interest
SBF: Serial blockface
SEM: Scanning electron microscope
