# Supplementary figures and images for: Hierarchical imaging: a new concept for targeted imaging of large volumes from cells to tissues
Source: BMC Cell Biol. 2016 Dec 12;17:38. doi: 10.1186/s12860-016-0122-8 (PMC5154069; doi:10.1186/s12860-016-0122-8)

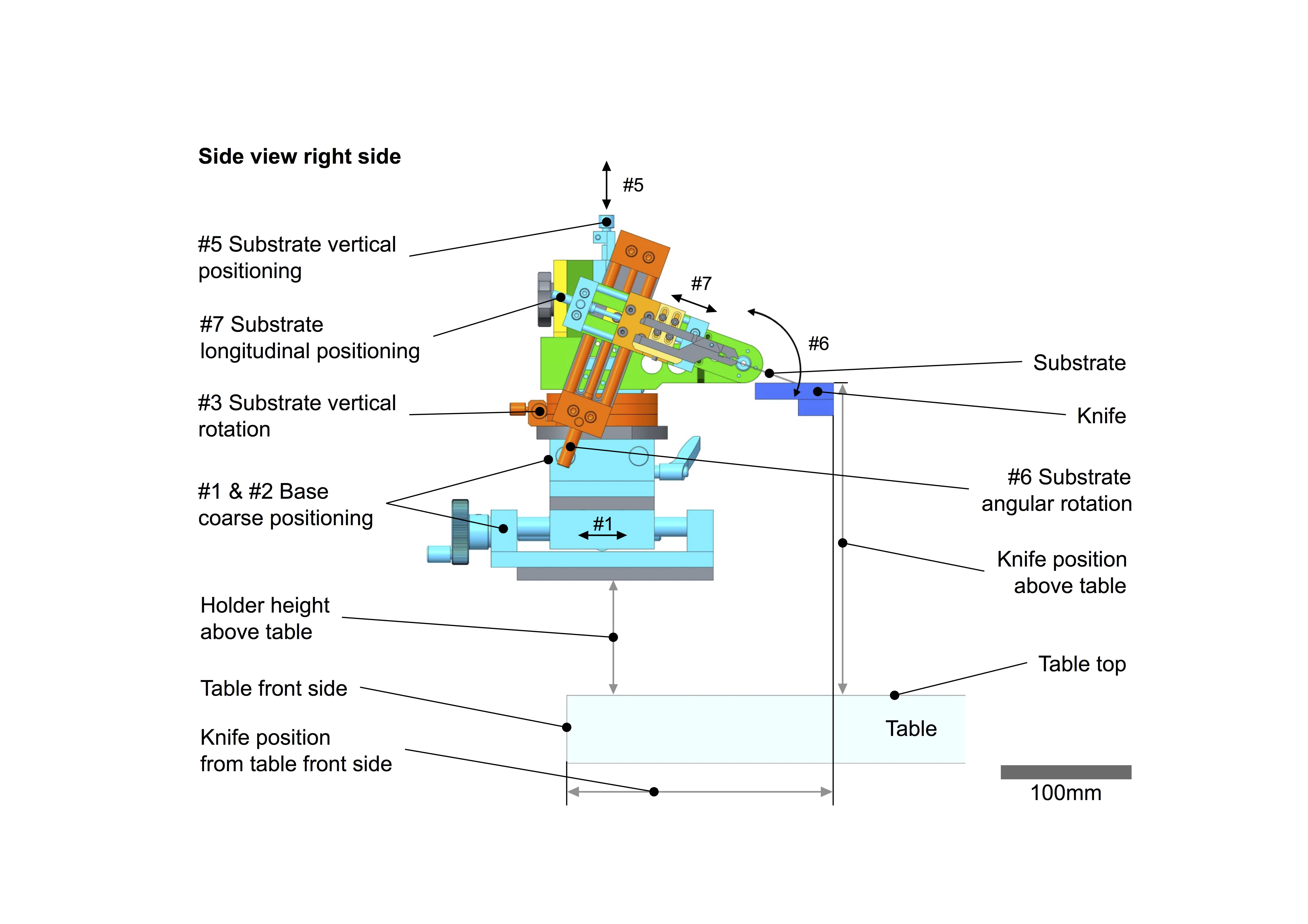

Supplement: Additional file 1: Figure S1. — 3D CAD model of substrate holder, side view right side (TIFF 3603 kb) [file 12860_2016_122_MOESM1_ESM.tiff]

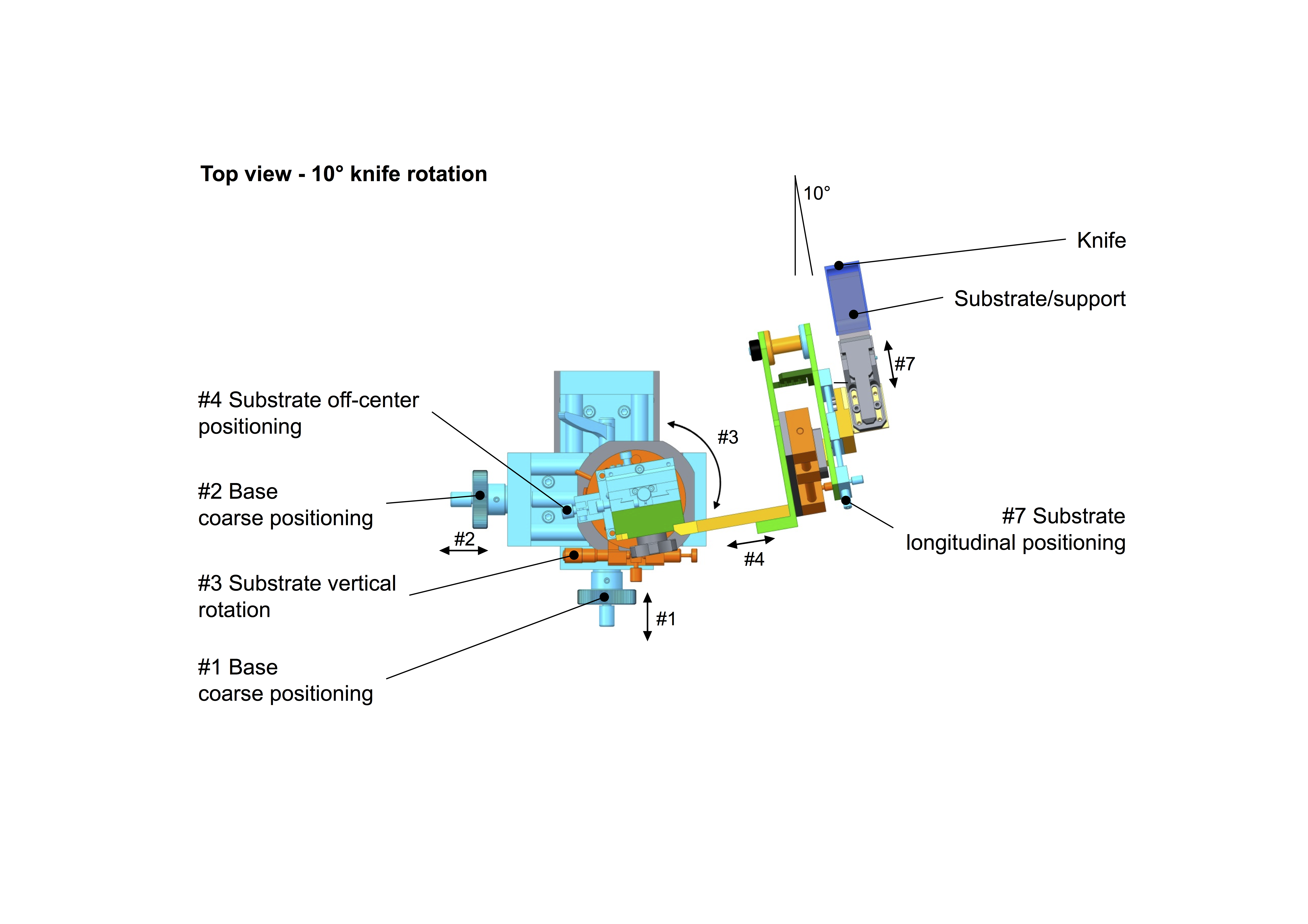

Supplement: Additional file 2: Figure S2. — 3D CAD model of substrate holder, top view – 10° knife rotation (TIFF 3524 kb) [file 12860_2016_122_MOESM2_ESM.tiff]

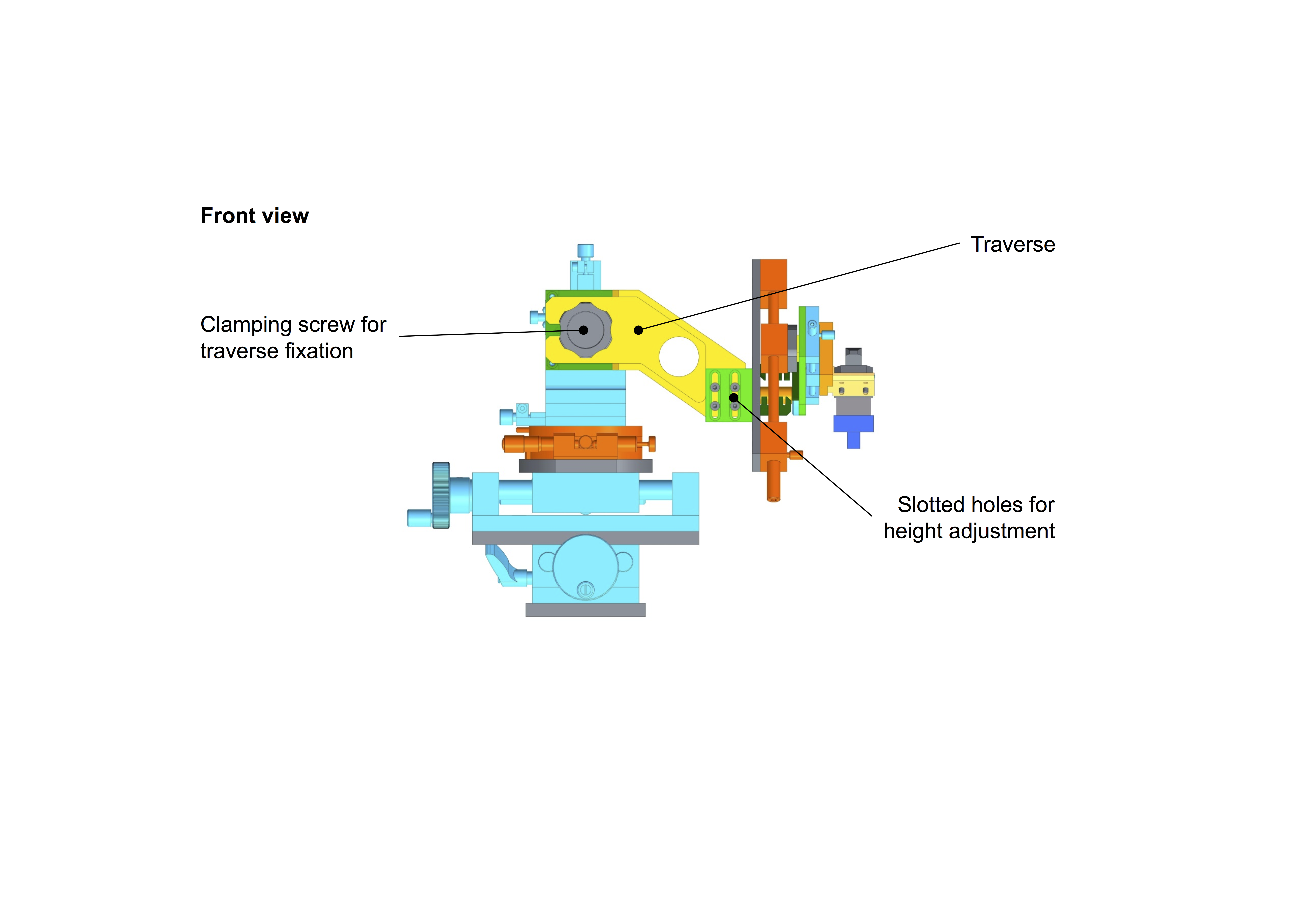

Supplement: Additional file 3: Figure S3. — 3D CAD model of substrate holder, front view (TIFF 3032 kb) [file 12860_2016_122_MOESM3_ESM.tiff]

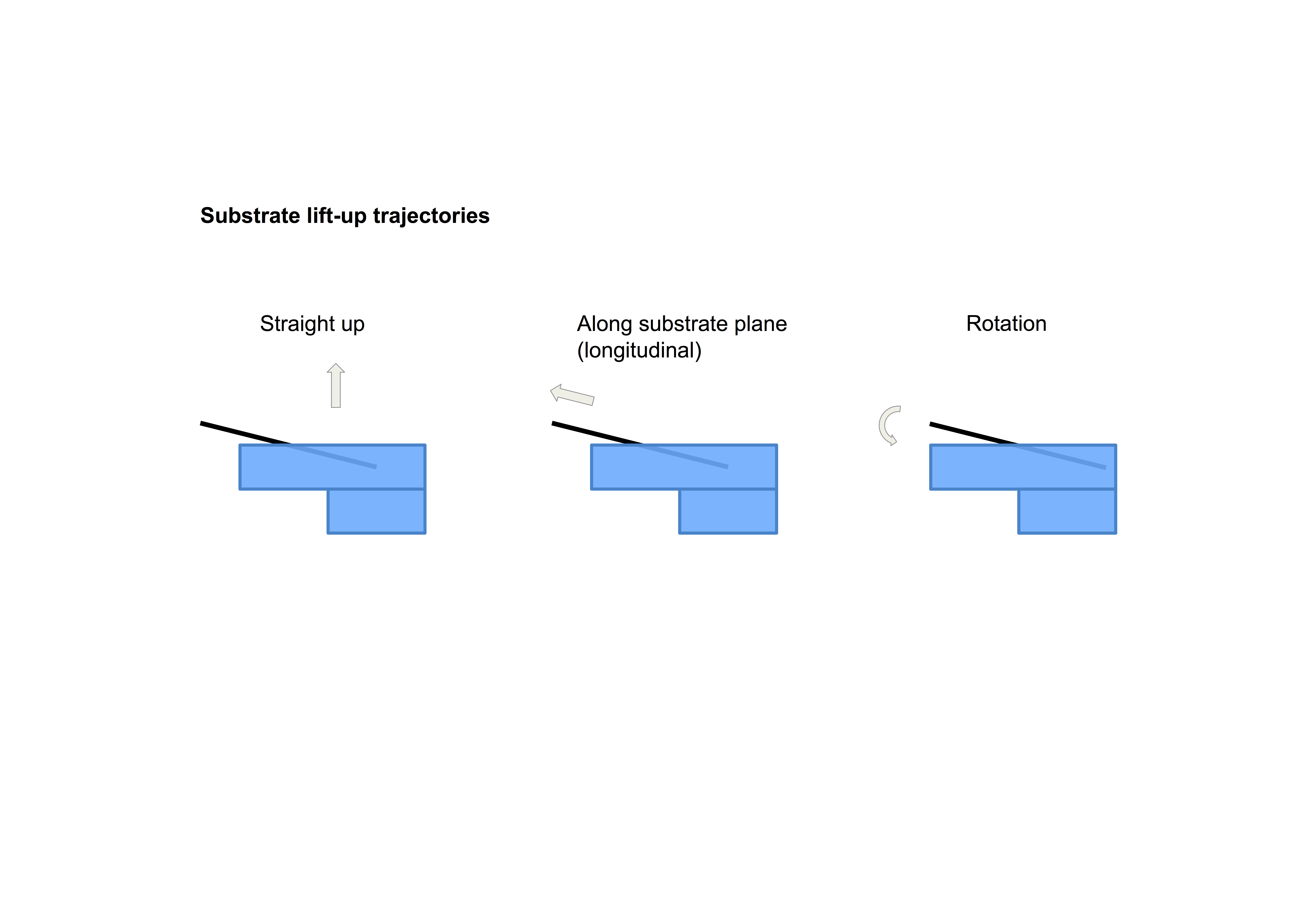

Supplement: Additional file 5: Figure S4. — Substrate lift-up trajectories (TIFF 915 kb) [file 12860_2016_122_MOESM5_ESM.tiff]
